# The Clinical Utility of Optical Genome Mapping for the Assessment of Genomic Aberrations in Acute Lymphoblastic Leukemia

**DOI:** 10.3390/cancers13174388

**Published:** 2021-08-30

**Authors:** Jonathan Lukas Lühmann, Marie Stelter, Marie Wolter, Josephine Kater, Jana Lentes, Anke Katharina Bergmann, Maximilian Schieck, Gudrun Göhring, Anja Möricke, Gunnar Cario, Markéta Žaliová, Martin Schrappe, Brigitte Schlegelberger, Martin Stanulla, Doris Steinemann

**Affiliations:** 1 Department of Human Genetics, Hannover Medical School, 30625 Hannover, Germany; Luehmann.Jonathan@mh-hannover.de (J.L.L.); stelter.marie@freenet.de (M.S.); mwolter97@web.de (M.W.); Kater.Josephine@mh-hannover.de (J.K.); Lentes.Jana@mh-hannover.de (J.L.); Bergmann.Anke@mh-hannover.de (A.K.B.); schieck.maximilian@mh-hannover.de (M.S.); goehring.gudrun@mh-hannover.de (G.G.); Schlegelberger.Brigitte@mh-hannover.de (B.S.); 2 Department of Pediatrics I, ALL-BFM Study Group, Christian-Albrechts University Kiel and University Medical Center Schleswig-Holstein, 24105 Kiel, Germany; a.moericke@pediatrics.uni-kiel.de (A.M.); gunnar.cario@uksh.de (G.C.); m.schrappe@pediatrics.uni-kiel.de (M.S.); 3 Department of Paediatric Haematology and Oncology, 2nd Faculty of Medicine, Charles University and University Hospital Motol, CZ-15006 Prague, Czech Republic; marketa.zaliova@lfmotol.cuni.cz; 4 Pediatric Hematology and Oncology, Hannover Medical School, 30625 Hannover, Germany; stanulla.martin@mh-hannover.de

**Keywords:** ALL, Optical Genome Mapping, molecular karyotyping, prognostic marker, risk assessment, gene fusion, copy number alteration

## Abstract

**Simple Summary:**

The stratification of childhood ALL is currently based on various diagnostic assays. This study investigates the feasibility of Optical Genome Mapping (OGM) to determine the genetic risk profile of ALL using fresh and frozen blood cells in an all-in-one approach. Acute lymphoblastic leukemia samples with data available from SNP-array/array-CGH, RNA-Seq, MLPA, karyotyping and FISH were compared to results obtained by OGM. We show that OGM has the potential to simplify the diagnostic workflow and to identify new structural variants helpful for classifying patients into treatment groups.

**Abstract:**

Acute lymphoblastic leukemia (ALL) is the most prevalent type of cancer occurring in children. ALL is characterized by structural and numeric genomic aberrations that strongly correlate with prognosis and clinical outcome. Usually, a combination of cyto- and molecular genetic methods (karyotyping, array-CGH, FISH, RT-PCR, RNA-Seq) is needed to identify all aberrations relevant for risk stratification. We investigated the feasibility of optical genome mapping (OGM), a DNA-based method, to detect these aberrations in an all-in-one approach. As proof of principle, twelve pediatric ALL samples were analyzed by OGM, and results were validated by comparing OGM data to results obtained from routine diagnostics. All genomic aberrations including translocations (e.g., dic(9;12)), aneuploidies (e.g., high hyperdiploidy) and copy number variations (e.g., *IKZF1*, *PAX5*) known from other techniques were also detected by OGM. Moreover, OGM was superior to well-established techniques for resolution of the more complex structure of a translocation t(12;21) and had a higher sensitivity for detection of copy number alterations. Importantly, a new and unknown gene fusion of *JAK2* and *NPAT* due to a translocation t(9;11) was detected. We demonstrate the feasibility of OGM to detect well-established as well as new putative prognostic markers in an all-in-one approach in ALL. We hope that these limited results will be confirmed with testing of more samples in the future.

## 1. Introduction

Acute lymphoblastic leukemia (ALL) is the most frequent cancer in childhood, with B-cell precursor ALL (BCP-ALL) accounting for the majority of cases. In Germany, around 500 pediatric BCP-ALL are newly diagnosed every year. Nowadays, nearly 90% of cases treated according to the BFM (Berlin-Frankfurt-Münster) protocols achieve long-term cure. In spite of the improved outcome, a significant proportion of patients relapse or experience severe therapy-related toxicities [1,2,3]. The aims to better understand cancer cell behavior and to further tailor treatment to individual patients require the precise determination of known genetic markers of relapse prediction and therapeutic decision making. Historically, the intensity of treatment is based on a patient’s risk to relapse, which is predicted by a combination of clinical (e.g., age, white blood cell count), cytogenetic, and morphological early response criteria. It has been shown that monitoring of leukemic blast clearance as minimal residual disease (MRD) reflects the treatment response and is the strongest generally applicable prognostic factor in pediatric ALL [4]. In addition to MRD, a broad spectrum of known underlying genetic aberrations is used for risk stratification. Markers for poor prognosis are gene fusions of *BCR/ABL1* as a result of the chromosomal translocation t(9;22), *TCF3/HLF1* due to the translocation t(17;19) as well as *KMT2A* fusions with different partner genes or the loss of whole chromosomes, which is called hypodiploidy (<45 chromosomes) [5,6,7,8]. In addition, there are also markers indicative for a favorable outcome, such as the *ETV6/RUNX1* fusion that results from a t(12;21) rearrangement [9,10]. Treatment intensity strongly depends on these genetic stratification markers [11].

Genome- and transcriptome-wide profiling has increased our understanding of the genetic basis of ALL enormously and has led to the identification of multiple genomic events that define new subtypes of ALL. A variety of genetic alterations that lead to gene fusions that are sensitive to tyrosine kinase inhibitors (e.g., *ABL1* and *PDGFRB* rearrangements) or JAK inhibitors (e.g., *JAK2*, *EPOR*, *IL7R* rearrangements) have been described in recent years for the group of *BCR/ABL1*-like ALL [12,13,14,15,16]. Copy number alterations, such as microdeletions and duplications, also play a role in treatment response [17]. Recently, we described a new prognostic subgroup called *IKZF1*^plus^ [18]. Several methods depending on dividing cells, DNA, and RNA are currently used to detect all relevant rearrangements. Karyotyping is still considered the gold standard to detect chromosomal aberrations. Optical genome mapping (OGM) has recently been introduced as an all-in-one technique that is able to detect balanced and unbalanced translocations, copy number variations in a range of few kb up to whole chromosomes (aneuploidies) as well as genomic insertions and inversions [19,20,21]. OGM is based on the imaging of labeled and linearized ultra-high molecular weight (UHMW) DNA. The images of labeled DNA molecules of each individual genome are converted into maps, which are then assembled into genome scaffolds. The comparison of patients’ genome assemblies to a reference genome assembly searching enables the identification of structural variants (SV) from discordant genome maps. Long-range genomic information is taken directly from the patients’ DNA in a single analysis without previous culture or enrichment of DNA. Recently, in a series of 52 AML samples comparing known cytogenetic abnormalities with OGM, multiple novel somatic events, including balanced translocations have been identified [22]. It was our aim to confirm that OGM is a suitable methodology to identify ALL-specific stratification markers from fresh and archived blood cells. Therefore, we tested five well-characterized ALL cases with the known *IKZF1*^plus^ profile retrospectively from banked cells as well as seven prospective samples from a routine ALL diagnostic workflow, which allowed a broad comparison of OGM to the full spectrum of other routinely used methods.

## 2. Materials and Methods

### 2.1. Isolation of Ultra-High Molecular Weight (UHMW) gDNA

All twelve patient samples were obtained at ALL diagnosis including seven AIEOP-BFM ALL 2017 (NCT03643276) blood samples, randomly taken from excess blood from diagnostic procedures, as well as five samples of human peripheral blood mononuclear cells (PBMCs) stored in liquid nitrogen with an *IKZF1^plus^* profile according to SNP-array or MLPA (retrospective analysis ALL-BFM 2000, NCT00430118). Informed consent was obtained from all subjects involved in the study. The samples are also part of other studies. The UHMW gDNA was isolated according to the manufacturer’s guidelines with small changes (Bionano Prep SP Frozen Human Blood DNA Isolation Protocol #30246, Bionano Genomics, San Diego, CA, USA and Bionano Prep Cell Culture DNA Isolation Protocol #30268, Bionano Genomics, San Diego, CA, USA). Briefly, cell counts were determined using HemoCue (Radiometer, Copenhagen, Denmark) and 2.5 × 10^6^ cells (instead of the suggested 1.5 × 10^6^ cells) from each sample were used for DNA isolation. After centrifugation the cell pellet was resuspended in DNA stabilizer and Proteinase K in lysis-buffer was added and the sample was rotated for 15 min on the HulaMixer (ThermoFisher Scientific, Waltham, MA, USA). After adding PMSF (Sigma-Aldrich, St. Louis, MO, USA) and incubation for 10 min, a Nanobind disk and isopropanol were added to each sample. To bind the gDNA to the disks, the samples were rotated on the HulaMixer for 15 min. Next, the disks were washed with buffers WP1 and WP2 and then transferred into new tubes. The DNA was eluted from the disks by incubating the disks in EB buffer. After incubation, the EB buffer was transferred to a new tube and the eluted DNA was mixed by pipetting and stored at room temperature to facilitate the homogenization of the DNA.

### 2.2. DNA Quantification

To quantify the gDNA the Qubit^TM^ dsDNA BR Assay Kit with a Qubit 3.0 Fluorometer (ThermoFisher Scientific, Waltham, MA, USA) was used. To ensure the homogeneity of the UHMW gDNA, the concentration was determined at three different points in the tube (left, middle and right side) The gDNA isolation was considered successful when the DNA concentration was equal or above 36 ng/µL and the coefficient of variation (CV) was <0.3.

### 2.3. Labeling of UHMW gDNA and Chip Loading

The labeling of the UHMW gDNA was done according to the manufacturer’s guidelines by using the Bionano Prep Direct Label and Stain (DLS) Protocol. Purified UHMW gDNA (750 ng) was first labeled with DL-green fluorophores using the DLE-1 enzyme. Next, Proteinase K digestion (Qiagen, Hilden, Germany) was performed and DLE was cleaned up using two different membranes on a microplate. The backbone of the DNA was labeled using DTT and DNA stain (DNA stain reagent, Bionano Genomics, San Diego, CA, USA). Then, the sample was rotated on the HulaMixer followed by overnight incubation at room temperature and protected from light. Two samples of each labeled gDNA taken from two different locations within the tube (left and right side) were quantified using the Qubit^TM^ dsDNA HS Assay kit (ThermoFisher Scientific, Waltham, MA, USA). The recommended DNA concentration is 4–12 ng/μL and a CV between the sampling <0.25. The labeled gDNA was loaded on the Saphyr G2.3 chips and molecules were imaged by the Saphyr instrument with a maximum capacity of 2000 Gb per sample.

### 2.4. Assemblies and Variant Calling

The Bionano Access software was used for the de novo assemblies and structural variant calling (Tools Version 1.5.2, Bionano Genomics, San Diego, CA, USA). After the molecule files of each sample were generated, the data was filtered down to 500 Gb with a minimal molecule length of 150 kb. From this filtered file a de novo assembly was done for each sample. The data were further filtered with the recommended confidence filter settings. The confidence level for inter-chromosomal translocation was set to 0.01. The frequency of SVs present in the Bionano control sample cohort was set to 0%. The self molecule count (minimum amount of molecules supporting the variant) was set to 10. As reference genome hg19 was used. To identify the origin of the inserted material the Rare Variant Pipeline (RVP; Tools Version 1.6.1, Bionano Genomics, San Diego, CA, USA) with a total throughput of 2000 Gb was used for patient 4. The clinical relevance for ALL of the structural variation of each called SV was then determined by manual inspection and comparison to data available from respective diagnostic reports or from the literature.

### 2.5. Data Comparison

The mapping results were compared to the known aberrations from karyotyping, FISH (Fluorescence in situ hybridization) analysis, array-CGH and RNA-panel sequencing (methods are described in [23]). Whole transcriptome sequencing (RNAseq) of diagnostic samples (in 5/12 patients, #1–5) was performed as a service at the Sequencing Core Facility of the Max Planck Institute for Molecular Genetics (Berlin, Germany) (Table 1). Sequencing libraries were prepared from DNA and total RNA using TruSeq kits (Illumina, San Diego, CA, USA) and sequenced on HiSeq2000 platform (Illumina, San Diego, CA, USA). The read pairs were aligned to the human genome reference hg19 using STAR (RNAseq) aligners and further processed by Picard tools (http://broadinstitute.github.io/picard/releases/tag/1.113 accessed on 29 August 2021). 

We focus here on called SVs and CNVs, which are known to be clinically relevant or which may be clinically relevant based on their frequency in reference genomes but we will not address polymorphic regions according to DGV (http://dgv.tcag.ca/dgv/app/home, release date 25 February 2021). A selected number of SVs called in Bionano, but undetected by standard methods were validated by breakpoint-spanning PCR or FISH.

## 3. Results

All twelve samples met the quality standards regarding cell count, DNA length, generated map counts and length. Notably, the quality of the analysis was not influenced whether frozen (patient 1–5) or fresh (patient 6–12) material was used for isolation of UHMW-DNA (Table S1). In total, >1.25 × 10^6^ molecules for each sample were aligned to the reference genome, resulting in an average effective coverage of the reference of 111×. The following results were obtained using the de novo assembly pipeline with appropriate filter settings for confidence and size to prioritize SV.

### 3.1. Patients 1–5 

**Figure 1:**
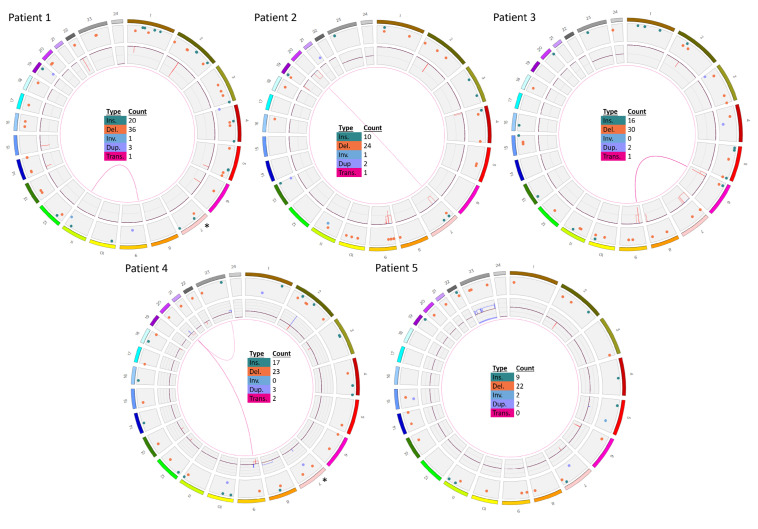
Genome-wide visualization of OGM data obtained from a retrospective analysis of five *IKZF1*del-positive samples. Circos plots show translocations between two chromosomes in the middle as lines from one chromosome to another. The inner ring gives copy number status (whole chromosome aneuploidies or partial intrachromosomal deletions and gains/amplifications. The next ring shows all other types of SV as colored dots: insertions in green, deletions in orange, inversions in blue and duplications in purple. The outer ring shows chromosomes from 1 to 24 (1 to 22, X,Y). Patients 1–5 according to Table 1 and Table 2 are shown. * The tiny *IKZF1* deletions in patients 1 and 4 were masked by the annotation software, therefore the corresponding dots were added manually to the circos plot.

**Figure 2:**
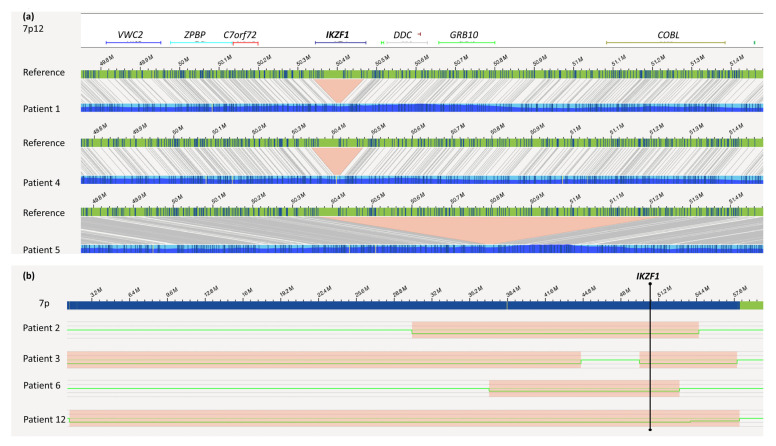
*IKZF1* deletions: as detected by OGM. (**a**) Small deletions affecting *IKZF1* within 7p12.2p12.1; the green bar indicates the reference map; patient maps are displayed in blue; deletions are marked in red. (**b**) Ideogram of 7p from ptel to cen, showing larger deletions (red boxes) within the 7p arm in patients 2, 3 and 6 (upper part), green lanes give the copy number along the 7 p-arm at 2n (normal copy number) or 1n (one deleted copy). All *IKZF1* aberrations were detected by the de novo assembly algorithm.

In five retrospective patient samples selected from ALL-BFM 2000 for their high risk and known *IKZF1*-deletion profile, OGM was able to confirm all *IKZF1* profiles (Table 1 and Table 2, Figure 1). The exact genomic sizes of all *IKZF1* deletions are shown in Figure 2.

**Figure 3:**
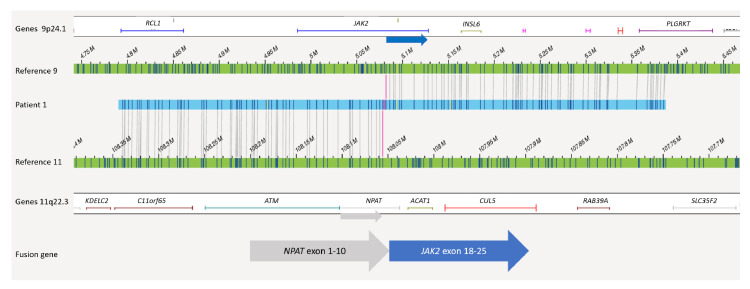
Balanced translocation t(9;11)(p24.1;q22.3) in patient 1 of OGM. The upper green bar shows the reference map of chr 9 and the lower green bar the reference map of chr 11. Genes located in the depicted regions are indicated above or below. In between, a map of patient 1 (blue) is shown where half of the labels fit to chr 9 and the other half fit to chr 11. Both breakpoints are marked with red lines hitting *JAK2* and *NPAT*.

According to OGM, the *IKZF1* deletion in patient 1 had a size of nearly 130 kb with intragenic *IKZF1* breakpoints in intron 3 and intron 7 (7p12.2(50339472_50467354) × 1). In agreement with SNP/MLPA data, no additional *IKZF1^plus^*-specific deletions (namely *CDKN2A/B*, *PAX5*, *CRLF2-P2RY8*) were detected in this case. Due to chromosome banding analysis, the karyotype was described as 46,XX,del(11)(?p21q23). Importantly, reanalysis of this sample by means of OGM led to the identification of a translocation t(9;11)(24.1;q22.1) involving the genes *JAK2* and *NPAT* (Figure 3). A possible gene fusion was indicated by OGM with a breakpoint on chr 9 between position 5079902 and 5081915 and a breakpoint on chr 11 between position 108046275 and 108055698. This could lead to a fusion of *NPAT* (exon 10) with exons 17, 18, 19, or 20 of *JAK2.* In fact, RNA-Seq confirmed a fusion gene by reads running from NPAT exon 10 into JAK2 exon 18. A detailed comparison of all available data is given in Table 1 and Table 2.

In patient 2, the known *IKZF1^plus^* profile from SNP/MLPA was confirmed by OGM indicating a mono-allelic deletion at 7p14.3p11 (*IKZF1*) and a deletion at 9p24.3p13.1 that included the *CDKN2A/B* locus, which was deleted bi-allelically. In addition, a deletion of 20q11.21q13.33 that resulted in the translocation t(7;20)(p14.3;q11.21) and a 6.5 Mb large deletion at 4p16.3p16.1(1217567_7779679) were detected by OGM.

Similarly, patient 3 carried an *IKZF1^plus^* profile, including deletions at 7p12.2p11.2 and 9p21.p13.1. Again, the *CDKN2A/B* gene locus was deleted on both alleles. Additional SVs were detected by OGM (e.g., a translocation t(6;9)(p22.3;p22.1) conducted by deletions at 6p25.3p22.3(76216_18127228) and at 9p21.3p13.1(22121006_38792295)). The translocation breakpoints were located intragenically of *TPMT* and *CDKN2B-AS1*, most likely leading to inactivation of both involved genes (Figure 1, Table 1 and Table 2).

In patient 4, the *IKZF1* deletion at 7p12.2(50345917_50475831) included exons 2–8. In addition to *IKZF1^plus^*, a dic(9;20)(p13.1;q11.23) and a translocation t(X;20)(q27.1;q11.22) were observed. These structural alterations included loss of 9p, a deletion at 20q11.23q13.33(37233950_62965071) and a duplication Xq27.1q28(138091547_155255277) (Figure S1). Additionally, the insertion (ins(12;9)(p13.2;q34.13)(12:1203481_12043458ins(9:133722840_133791173)) that resulted in an *ETV6*/*ABL1* fusion was identified. *ABL1* exons 2–11 are thereby juxtaposed downstream of *ETV6* exon 5 (Figure S2).

In patient 5, the *IKZF1^plus^* profile consisted of an *IKZF1* deletion at 7p12.2p12.1 (50364101_51250510) showing intragenic breakpoints at *IKZF1* (intron 2) and *COBL* (intron 4) in addition to an intragenic *PAX5* deletion (9p13.2(36846196_36969903)). Consistent with SNP-array data, a gain of chromosome X was also detected by OGM. 

### 3.2. Patients 6–12

**Figure 4:**
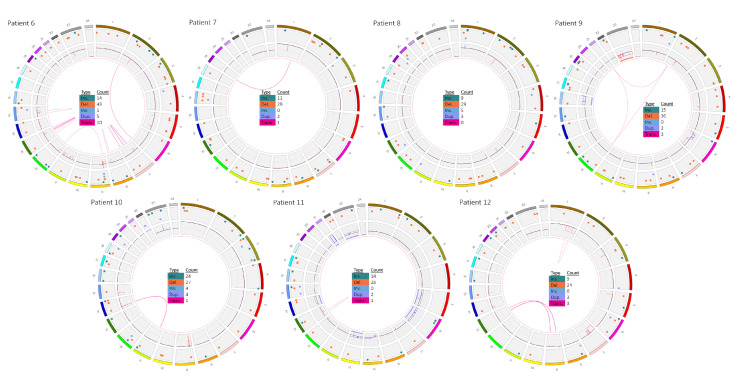
Genome-wide visualization of OGM data obtained from a analysis of seven prospective ALL samples. Circos plots show translocations between two chromosomes in the middle, as line from one chromosome to another one. The inner ring gives copy numbers (whole chromosome aneuploidies or partial intrachromosomal deletions (these ones are also shown as intrachromosomal translocations) and gains/amplifications). The next ring shows all other kinds of SV as colored dots: insertions in green, deletions in orange, inversions in blue and duplications in purple, the outer ring shows chromosomes from 1 to 24 (1 to 22, X,Y). Samples 6–12 according to Table 1 and Table 2 are shown.

The OGM circos plots of samples 6 to 12 (AIEOP-BFM ALL 2017) are given in Figure 4.

**Figure 5:**
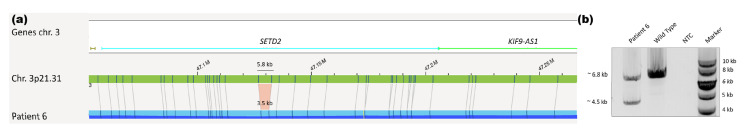
(**a**) Heterozygous *SETD2* deletion by OGM. The green bar displays the reference map, the blue/turquois bar the map of patient 6. The distance between the labels at positions 47,126,654 and 47,132,458 in the reference was 5.8 kb, whereas in the patient the distance was 3.5 kb, thus indicating a 2.3 kb deletion. (**b**) Validation of the *SETD2* deletion via PCR. In the wild type, one band at 6.8 kb was observed, while in the patient sample an additional band at 4.5 kb containing the deletion was present. NTC: no template control.

The most complex alterations were detected in patient 6, whose ALL cells were also *IKZF1^plus^* positive. Chromosome analysis determined the karyotype as 44,XX,-7,dic(9;12)(p13;p13),-11,del(15)(q21),+mar1~2[15]/46,XX[5]. Array-CGH showed that only the p-arm of chromosome 7 was lost and that additional deletions were present (Table 1). No gene fusions were detected by RNA-Seq. OGM also detected all of these alterations as well, not only the large number of deletions with very similar breakpoints compared to array-CGH, but also the translocation dic(9;12)(q21.1;q12.1) that resulted from 9p and 12q losses. In accordance with RNA-Seq, no gene fusion was indicated by OGM. Importantly, additional microdeletions below the resolution of array-CGH were detected, including an intragenic *SETD2* deletion (chr3: 47126654_47132458) with a size of around 2.3 kb, which was confirmed by breakpoint-spanning PCR (Figure 5).

In patient 7, OGM showed a translocation t(1;19)(q23;p13) with fusion of *TCF3* and *PBX1* and a bi-allelic loss of 9p21.3 including *CDKN2A/B*. The *TCF3/PBX1* fusion was resolved by chromosome banding and RNA-Seq as well, whereas the *CDKN2A/B* deletion was confirmed by array-CGH. Additional findings were not observed in this patient using OGM.

A good concordance in SV detection between OGM and array-CGH was also true for patient 8. Both methods detected a bi-allelic *CDKN2A/B* deletion and a bi-allelic deletion at 13q14 (*RB1*) (Figure S3). Of note, use of OGM enabled discrimination between both *RB1* alleles, which showed one allele with a loss ranging from 48,981,801 to 49,084,431 and the other allele with a loss ranging from 48,983,012 to 49,148,801.

**Figure 6:**
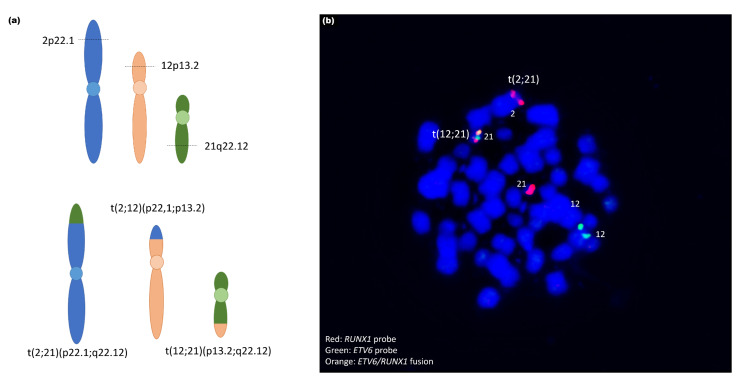
(**a**) Schematic representation of three-way translocation.t(2;12;21) (p22.1;p13.2;q22.12) ogm[hg19]g [chr2:pter_39472511::chr12:12034841_cen_qter]g.[chr12:pter12023465::chr21:36256442_cen_pter]g.[chr21:qter36277853::chr2:39474805_cen_qter. (**b**) Validation of the translocation by metaphase-FISH. Hybridization with ETV6/RUNX1-probe. Red: *RUNX1*-signal; Green: *ETV6*-signal; Orange: Fusion signal *RUNX1*/*ETV6*. The *RUNX1*-signal is visible on chr 2.

In patient 9, chromosome banding showed an *ETV6/RUNX1* translocation t(12;21). Interestingly, OGM revealed that this translocation was more complex and additionally involved parts of chromosome 2. In fact, a three-way translocation t(2;12;21)(p22.1;p13.2;q22.12) was confirmed via FISH using the *ETV6/RUNX1* probe (Figure 6). Furthermore, chromosome 6 showed a complex CNV profile, including two deleted parts at the ends of the p- and q-arms and duplication of the large middle part as well as one X-chromosome loss.

OGM data for patient 10 indicated the diagnosis of a T-ALL (Table 1 and Table 2). A *TAL1*/*STIL* deletion restricted to malignancies of the T cell lineage at 1p33 was present as well as a TCR rearrangement t(11;14) targeting *CAPRIN1* at 11p13 and the *TCRA* at 14q11.2.

In patient 11, OGM and array-CGH results were very comparable, showing a high hyperdiploid karyotype with gains of chromosomes +5, +6, +10, +14, +21(x~6), +X. Furthermore, a loss of 9p24.3p13.1 and a gain of 9q22q34.2 were indicated by genome maps. The 9p deletion on one allele and a small deletion at 9p21.3(21678074_22273253) on the other allele led to a bi-allelic loss of *CDKN2A/B* in this case. Next to the aberrant chromosome 9, a dicentric chromosome 13, dic(13;13)(q14.13;q12.11) with fusion of *ZMYM2/ESD* was indicated and confirmed by RNA-Seq.

Patient 12 showed two large interstitial deletions within 1q (from 1q25.1 to 1q32.1 and from 1q41 to 1qter). An inversion of the region 1q32.1q41 that led to the juxtaposition of the genes *SLC9C2/KCTD3* was detected. Chromosome 7 showed a large deletion from 7pter to 7q21.11 including *IKZF1* (Figure 2). In addition, t(1;13)(q23.1;q22.3) targeting the genes *MIR181A1HG* and *SLAIN1* was present next to two translocations that both involved chromosomes 9 and 13: a t(9;13)(p13.2;q14.11) with breakpoints targeting *ZCCHC7* and *TSC22D1* and a t(9;13)(q31.2;q22.3) with no intragenic breakpoints. Accordingly, no fusions were observed by RNA-Seq.

## 4. Discussion

Since the genetic background of ALL impacts the initiation, progression and outcome of this disease, it is of utmost importance to determine chromosomal alterations in diagnostic samples as precisely as possible to better understand the molecular basis of treatment response. The adjustment of new and personalized treatment regimens to patients carrying genomic alterations that indicate poor response or are druggable by TKI will help to prevent treatment toxicities or disease relapses. However, solving the genomic structure of ALL is costly and time-consuming and includes numerous technologies and different patient materials. With the aim to test the feasibility of OGM in the genetic classification of pediatric ALL, we selected genetically well-characterized ALL samples, which were intensively analyzed by well-established technologies (SNP-array, RNA-Seq, FISH, karyotyping, MLPA) used within our routine diagnostic workflow. Recently, it was shown that OGM may have the potential to be a routine tool in malignant hematology [24]. We show here for the first time that OGM allowed the detection of all chromosomal stratification-relevant genomic markers in ALL, including translocations, aneuploidies, and copy number profiles including *IKZF1^plus^* in a retrospective as well as in a prospective manner.

### 4.1. IKZF1 Deletion

All *IKZF1*, as well as IKZF1plus deletions known from array-CGH/SNP analysis ranging from small intragenic to whole 7p deletions, were depicted in optical maps (patients 1–6, 12; Figure 2). Importantly, some of the patients with *IKZF1* deletions/*IKZF1^plus^* profiles showed additional alterations known to confer poor prognosis *per se*.

### 4.2. Translocations and Fusions

Importantly, an undescribed *JAK2* fusion partner, *NPAT,* was detected due to the translocation t(9;11) in one patient. This may be a treatment-relevant finding since *JAK2*-kinase rearranged ALL with fusions of *JAK2* to *ATF7IP*, *EBF1*, *PAX5*, *SSBP2*, *RNPC3, GOLGA5, PCM1* and their effective inhibition by treatment with the JAK inhibitor ruxolitinib have been described previously in anecdotal cases [25,26,27,28]. Preclinical data suggested that ruxolitinib may be effective in BCR-ABL1-like ALL [29].

There is an ongoing discovery of new Ph-like alterations from unbiased genetic testing underlining the necessity for the implementation of this knowledge into clinical trials. However, our patient received stem cell transplantation and showed complete remission of the disease. Typical JAK2 fusions include the N-terminus of partner protein and the C-terminus of JAK2 including JH2 and/or JH1 domains. The *NPAT/JAK2* is a new fusion that has not been published previously. Its presence was confirmed by whole transcriptome sequencing. The fusion seems to be very rare since no additional cases were observed by RNA-Seq of approximately 1200 cases within the AIEOP-BFM ALL 2017 study so far.

Moreover, the gene fusions *TCF3/PBX1*, *ZMYM2/ESD1, ETV6/RUNX* were detected in this cohort by OGM as well as by other methods. Interestingly, the t(12;21) was shown to be more complex, involving parts of chromosome 2 (t(2;12;21)) and a breakpoint on chromosome 2p22.1 next to *MAP4K3* (Figure 6). However, we cannot conclude that there is deregulation of this gene due to this juxtaposition. To our knowledge, involvement of MAP4K3 in the pathobiology of ALL has not yet been described. The same patient (#4) displayed an insertion of a part of *ABL1* in the *ETV6* locus that led to a gene fusion. Leukemia with *ETV6*/*ABL1* fusions is rare and associated with a poor prognostic outcome, but are sensitive to tyrosine kinase inhibitors [30]. Notably, this cryptic insertion is frequently missed by FISH when using either *BCR/ABL1* or *ETV6/RUNX1* probes [31].

Some additional translocations (t(7;20), t(X;20), t(2;6)) detected by OGM do not seem to play a role in the context of leukemia, since the breakpoints lie outside of coding or gene-related regions.

Furthermore, a translocation t(6;9)(p22.3;p22.1) with breakpoints targeting *TPMT* on chromosome 6 and *CDKN2A-AS* on chromosome 9 was detected in one patient (#3, no karyotype data were available). This translocation t(6;9) and/or deletions at 6p/6p22.3 have not been described in ALL. The clinical relevance of this alteration, therefore, remains unclear. Interestingly, low activity polymorphisms in *TPMT* involved in the regulation of the thiopurine pathway and metabolism of 6-mercaptopurine, are risk factors for ALL and strongly influence drug efficacy and clinical toxicity [32,33,34]. It remains to be shown whether the somatic rearrangement leads to loss of function of TPMT and plays a role in therapy response and clinical outcome.

OGM led to diagnosis of one patient (#10) as T-ALL with a T-cell receptor α rearrangement, a t(11;14) as well as a typical deletion at 1p33(47693608_47790165)x1 that targeted the *TAL1/STIL* locus. Both alterations were previously detected by means of MRD marker analysis (Table 1).

### 4.3. Dicentric Chromosomes

A dicentric chromosome dic(9;20)(p13.1;q11.23) was present in one *IKZF1^plus^* patient (#4, Figure S3). The dic(9;20) with unbalanced nature is known to be present in ~2%–5% of pediatric BCP-ALL, depending on the detection method used (chromosome banding or FISH) [35,36,37]. This is considered a driver event in ALL, and often involves *PAX5* as the key genetic target and has been associated with poor prognosis [38,39]. In our patient, the deletion/fusion breakpoint is downstream of *PAX5* that led to its deletion.

In agreement with conventional cytogenetics, a dic(9;12) was observed by OGM. Importantly, breakpoints of the dic(9;12)(p13;p13) could be determined to (p21.1;p12.1), with position 29,314,194 on chromosome 9 and 25,884,653 on chromosome 12 leading to an exclusion of a *PAX5/ETV6* fusion [40], which was suggested from cytogenetic analysis and RNA-Seq as well. A dic(13;13)(q14.13;q12.11) with fusion of *ZMYM2/ESD* was detected by OGM (patient #11) and confirmed by RNA-Seq. Rare cryptic *ZMYM2/FLT3* fusions that led to the activation of the FLT3 kinase were described in myeloproliferative neoplasia and adult ALL [41,42], whereas *ESD* coding for an esterase has not been previously described as a fusion partner. From the present data, we cannot conclude if this enzyme becomes activated due to the fusion.

### 4.4. CNV Resolution

Our OGM data not only led to the identification of new alterations and validation of previous findings, but also to the more precise refinement of alterations known from routine diagnostics. OGM exhibited superior resolution compared to array-CGH, FISH and karyotyping. The smallest deletion we found in this cohort was a previously undetected *SETD2* deletion of 2.3 kb in one patient (#6), which was validated via PCR (Figure 5). Notably, deletions targeting *SETD2* are present in around 2% of ALL according to our AIEOP-BFM ALL 2017 in-house database of 1252 ALL cases analyzed by array-CGH so far. SETD2 is the sole methyltransferase responsible for cellular H3K36 tri-methylation in humans and plays a role in numerous cellular processes, including regulation of transcription, DNA replication and DNA repair after damage. Alterations such as focal deletions or missense variants in *SETD2* have been described in several cancers (reviewed in Skucha et al. 2019) [43]. Moreover, a potential role of *SETD2* in chemotherapy resistance has been discussed since alterations in this gene were found to be significantly enriched in relapsed pediatric ALL patients [44].

### 4.5. Advantages and Limitations of OGM

We show that OGM enables the genome-wide detection of different types of SVs in an all-in-one approach in ALL samples. OGM is based on the direct labeling of total DNA isolated from blood cells without culturing, thus omitting the potential selection of outgrowing clones. Since DNA has to be ultra-long archived DNA samples isolated with standard protocols can not be used because the fragments are too short. The quality of UHMW-DNA prepared from whole blood or frozen cells was high and reproducible and strongly dependent on the compliance with exact cell numbers used for preparation. Genomic rearrangements detectable by OGM may lead to gene fusions or differential gene expression. Therefore, RNA sequencing will be helpful to confirm and further characterize those cases.

Noteworthy, OGM is not a sequencing technique; hence, no information on bp level is obtained, meaning exact breakpoints, SNPs or small indels are not addressed. The price per sample ranges between USD 300 and 500, depending on the number of kits purchased, and is comparable to array-CGH. In contrast, the OGM workflow of around 5–7 days from sample acquisition to result, a few days longer than karyotyping or array-CGH.

The detection resolution for SVs mainly depends on the label distribution (~15 labels/100 kb) and can be as low as some hundred bp due to the calculation of label distances. A limitation of OGM is the detection of whole arm translocations (Robertsonian translocations) and SVs located in telomeric regions due to the absence of labels in these regions Depending on the amount of data generated (up to 4000 Gb), detection of SVs with allele frequencies below 10% may be possible [24]. Similar to observations by Lestringant et al., we were not able to detect *CRLF2/P2RY8* fusions in patients #2 and #5 because of subclonality (#2) and the repetitive nature of the PAR region at Xp22.33 [24]. To integrate OGM into the routine diagnostic workflow and on a larger scale, further validation and optimization of the workflow (e.g., increasing computing capacity and automated sample preparation to reduce processing time) is required.

## 5. Conclusions

We demonstrate that OGM can be used to identify novel genomic alterations in ALL and may help to redefine the established prognostic subgroups and to provide a better understanding of the pathobiology and molecular biology of treatment response. In regard to retrospective studies, OGM enables the analysis of SVs and gene fusions with a single method.

## Figures and Tables

**Table 1:** Results of conventional cytogenetic methods for patient 1–12.

Patient	Karyotype	Fish	Array-CGH/SNP-Array/MLPA ^1^	RNA-Seq Fusion
1	46,XX,del(11)(?q21q23)	*BCR*/*ABL*, *ETV6*/*RUNX1* and *MLL* negative	(*ARHGAP24*)x1,(*EBF1*)x1,(*IKZF1*)x1,(*SERP2*)x1, (*SLX4IP*)x1	*NPAT*/*JAK2*
2	-	*BCR*/*ABL*, *ETV6*/*RUNX1* and *MLL* negative	(*IKZF1*)x1,(*CDKN2A*)x0,(*PAX5*)x0	No fusion ^2^
3	-	*BCR*/*ABL*, *ETV6*/*RUNX1* and *MLL* negative	(*IKZF1*)x1,(*CDKN2A*)x0,(*CDKN2B*)x0,(*PAX5*)x1	No fusion
4	-	*BCR*/*ABL*, *ETV6*/*RUNX1* and *MLL* negative	(*FHIT*)x1,(*IKZF1*)x1,(*CDKN2A*)x0,(*CDKN2B*)x1, (*PAX5*)x1,(*BTG1*)x1,(*ATP10A*)x1,(*SLX4IP*)x1	No fusion
5	-	*BCR*/*ABL*, *ETV6*/*RUNX1* and *MLL* negative	(*IKZF1*)x1,(*PAX5*)x1,(Xp22.33)x1,(X)x3	No fusion ^2^
6	44,XX,-7,dic(9;12)(p13;p13),- 11,del(15)(q21),+mar1~2[15] /46,XX[5]	nuc ish 12p13(*ETV6*x1),21q22(*RUNX1*x2)[97/100],11q2 3(*MLL*x1)[96/100]	arr[GRCh37] 3q25.1(152025150_152078949)x1,7p14.2p12.1(36862295_5292 0052)x1,8q23.1q24.21(110045943_128549318)x1,9p24.3p21.1 (205069_29299751)x1,9p13.3p13.2(35259627_38394815)x1,10q 23.31(89650155_89697232)x1,11p15.1q24.2(20291195_127532 511)x1,12p13.33p12.1(162848_25877293)x1,15q13.2q22.1(303 66065_59117244)x1,18q21.2(52973141_53080556)x1,Xp11.3(4 4783685_44852481)x1	No fusion
7	46,XY,t(1;19)(q23;q13),inc[4] /46,XY[5]	9q34(*ABL1*x2),22q11(*BCR*x2)[97/100],12p13 (*ETV6*x2),21q22(*RUNX1*x3~4)[12/100],11q23 (*MLL*x2)[100/100],19p13(*TCF3*x2)(5′*TCF3*sep3′*TCF3*x 1)[90/100]	arr[GRCh37] 9p21.3(21986358_21993460)x0	*TCF3*/*PBX1*
8	46,XX[25]	nuc ish 8q24(*CMYC*x2)[99/100],9q34(*ABL1*x2),22q11 (*BCR*x2)[100/100],11q23(*MLL*x2)[100/100],12p13 (*ETV6*x2),21q22(*RUNX1*x2)[98/100],19p13 (*TCF3*x2)[97/100]	arr[GRCh37] 9p21.3(21938547_21993460)x0,13q14.2(48984925_49065340)x0	No fusion
9	-	12p13(*ETV6*x2~4),21q22(*RUNX1*x3~6)(*ETV6* con*RUNX1*x1~2)[100/100]	-	-
10	-	nuc ish 9q34(*ABL1*x2),22q11(*BCR*x2)[100/100],11q23 (*MLL*x2)[100/100] ^3^	-	-
11	-	nuc ish 9q34(*ABL1*x3),22q11(*BCR*x2)[52/100],12p13 (*ETV6*x2),21q22(*RUNX1*x4~5)[13/100],12p13 (*ETV6*x2),21q22(*RUNX1*x6)[29/100],12p13(*ETV6*x2), 21q22(*RUNX1*x7)[54/100],19p13(*TCF3*x2)	arr[GRCh37] 5p15.33q15(26142_95134494)x3,5q22.1q35.3(110817009_1806 96806)x3,6p25.3q27(204009_170911240)x3,9p21.3(21689983_ 22268884)x0,9p24.3p13.1(205069_38805758)x1,9q21.11q34.3 (70984481_141115978)x3,10p15.3q26.3(102539_135434178)x3, 13q14.2q14.3(47355214_53035715)x1,14q11.1q32.3(19043189 _106552084)x3,21q11.2q22.3(14539679_48090317)x~6, Xp22.33q28(204448_106552084)x3	*ZMYM2*/*ESD*
12	45,XY,del(1)(q25),del(7) (p11),add(9)(q21),del(9)(p12), -13,add(15)(p13)[4]// 46,XY[11]	nuc ish 9q34(*ABL1*x2),22q11(*BCR*x2)[97/100],11q23 (*MLL*x2)[98/100],12p13(*ETV6*x2),21q22 (*RUNX1*x2)[98/100],19p13(*TCF3*x2)[92/100]	arr[GRCh37] 1q25.1q44(173487058_249218792)x1,7p22.3p11.2(45130_5757 7373)x1,7q11.21q21.11(61821749_81088370)x1,15q12(260538 34_26096681)x1	No fusion

^1^ Only clinically relevant aberrations and not all detected CNV’s are listed. ^2^ Subclonal CRLF2/P2RY8 fusion detected by RT-PCR only. ^3^ MRD targets: SIL-TAL/db1, Db1-Jb2.7 indicating T-cell receptor rearrangement.

**Table 2:** Comparison of conventional cytogenetic diagnostic results with OGM results.

Patient	Results Validated by OGM ^1^	Novel Findings ^1^
1	ogm[hg19] 4q21.23(86445426_86506057)x1,5q33.3(157949400_158533971)x1,7p12.2(50339 472_50467354)x1,13q14.11(44845663_45020217)x1,20p12.2(10409635_1045849 8)x1	ogm[hg19] 46,XX,t(9;11)(p24.1;q22.3) *NPAT*-*JAK2* fusion ^2^: t(9p24.1;11q22.3)(5081915;108055699)
2	ogm[hg19] 7p14.3p11.2(30305758_54598807)x1,9p24.3p13.1(14566_39639975)x1,9p21.3 (21805860_22082060)x0,9p13.2(36841196_36892517)x0,20q11.21q13.33 (31411836_62965071)x1,Xp22.33(61554_1595639)x0~1	ogm[hg19] 46,XY,4p16.3p16.1(1217567_7779679)x1,t(7;20)(p14.3;q11.21)
3	ogm[hg19] 7p12.2p11.2(49589366_57834044)x1,9p21.3(21254326_22118700)x0,9p21.3p13. 1(22121006_38792295)x1	ogm[hg19] 46,XY,6p25.3p22.3(76216_18127228)x1,t(6;9)(p22.3;p21.3), 6p22.3(18127932_18519285)x0,7p22.3p13(10487_44611328) x1
4	ogm[hg19] 3p14.2(59995117_60552466)x1,7p12.2(50345917_50475683)x1,9p24.3p13.1(145 66_38598581)x1,9p21.3(21771311_22031191)x0,12q21.33(92168916_92587734) x1,15q12(25998721_26114333)x1,20p12.2(10409635_10458498)x1	ogm[hg19] 46,XX,8q13.19q24.1(66365779_144244501)x3,dic(9;20)(p13. 1;q11.23),9p13.1(38633406_39450127)x3,9p12(41639858_42 518127)x3,ins(12;9)(p13.2;q34.13)^3^,20q11.22q11.23(3277215 5_37232954)x3,20q11.23q13.33(37233950_62965071)x1,t(X; 20)(q27.1;q11.22),Xq27.1q28(138091547_155255277)x3 *ETV6-ABL* fusion ^2,3^: ins(12;9)(p13.2;q34.13)(12:12034841_12043458ins(9:133722 840_133791173)
5	ogm[hg19] 47,XX,7p12.2p12.1(50364101_51250510)x1,9p13.2(36846196_36969903)x1,Xp2 2.33(61554_2272814)x0~1,Xp22.33(61554_2274814)x0~2,Xp22.33q28(2674971_ 155255277)x3	*-*
6	ogm[hg19] 3q25.1(152014253_152086683)x1,dic(9;12)(q21.1;q12.1),7p14.1p12.1(36828512 _52954130)x1,8q23.1q24.21(110041918_128585274)x1,9p24.3p21.1(14566_2931 6907)x1,9p13.3p13.1(35227955_38397240)x1,10q23.31(89646060_89698668)x1, 11p15.1p11.12(83377_127712)x1,11q14.1q24.2(85137702_129511096)x1,12p13. 33p12.1(153001_25898742)x1,15q13.2q22.1(30356036_59124264)x1,18q21.2(52 966023_53084105)x1,Xp11.3(44782158_44859857)x1	ogm[hg19] 46,XX,t(2;6)(p24.3;q27),3p21.31(47126654_47132458)x1^2^,3q 22.3(136418158_136446074)x1,6p22.3(18232924_18249474) x1 ^4^
7	ogm[hg19] 46,XY,t(1;19)(q23;p13),9p21.3(21985616_21996138)x0 *TCF3*/*PBX1* fusion: t(1q23;19p13)(164755447;1638015)	-
8	ogm[hg19] 46,XX,9p21.3(21937565_21996138)x0	ogm[hg19] 13q14.2(48981801_49084431)x1,13q14.2(48983012_4914880 1)x1
9	ogm[hg19] t(12;21)(p13.2;q22.12)	ogm[hg19] 45,X,t(2;12;21)(p22.1;p13.2;q22.12)^4^,6p25.3p22.3(76216_168 28004)x1,6p22.1q14.1(16834818_79718301)x3,6q14.1q27(95 680530_170272658)x1,Xp22.33q28(61554_155255277)x1
10	ogm[hg19] 1p33(47693608_47790165)x1	ogm[hg19] 46,XY,9p24.1p21.2(14566_21574877)x1,9p24.1p21.2(215748 77_26276439)x0,9p13.3p13.1(33910882_39639975)x1,10q23 .31(89614306_89733606)x1~2,10q23.31(89688942_8923951) x1,t(11;14)(p13;q11.2)
11	ogm[hg19] 57,XX,5p15.33q15(1645821_95111596)x3,5q22.1q35.3(110834925_180899715)x 3,6p25.3q27(1851037_170272658)x3,9p24.3p13.1(14566_39639975)x1,9p21.3(2 1678074_22273253)x0,9q22q34.2(67985208_68496607)x3,10p15.3q26.3(64453_ 134158816)x3,13q14.2q14.3(47339725_53082621)x1,14q11.1q32.33(20526493_1 04540474)x3,21q11.2q22.3(14355427_46681324)x~6,Xp22.33q28(2663909_1548 80528)x3 *ZMYM2*/*ESD* fusion: dic(13;13)(q14.13;q12.11)(47355206;20574490)	-
12	ogm[hg19] 46,XY,1q25.1q32.1(173476173_215743425)x1,1q41q44(215736722_249237532)x1, 7p22.3q21.11(1307750_81073681)x1,15q12(26031156_26114333)x1	ogm[hg19] t(9;13)(p13.2;q14.11),ins(9;13)(q31.2;q22.3q14.11)?,t(13;15)? (q22.3;p13) ^4^

^1^ Not every CNV (known from DGV; http://dgv.tcag.ca/dgv/app/home, release date 25 February 2021) is listed. ^2^ Results were validated by independent methods. ^3^ Origin of inserted material was identified using RVP. ^4^ Most likely karyotype according to karyotyping and OGM.

## Data Availability

The datasets generated and/or analyzed during the current study are not publicly available but are available from the corresponding author on reasonable request.

## Supplementary Materials

The following data are available online at https://www.mdpi.com/article/10.3390/cancers13174388/s1, Figure S1: Translocation t(X;20) and t(9;20) in patient 4, Figure S2: Ins(12;9) leading to *ETV6*-*ABL1* fusion, Figure S3: Bi-allelic deletion in *RB1* in patient 8, Table S1: Sample description, OGM quality data and description of individual structural variants after de novo assembly.
