# Supplementary material for: The Clinical Utility of Optical Genome Mapping for the Assessment of Genomic Aberrations in Acute Lymphoblastic Leukemia
Source: Cancers (Basel). 2021 Aug 30;13(17):4388. doi: 10.3390/cancers13174388 (PMC8431583; doi:10.3390/cancers13174388)
Supplement: Supplementary file 1 [file cancers-13-04388-s001.zip › cancers-1327634-supplementary.pdf]

Supplementary Material

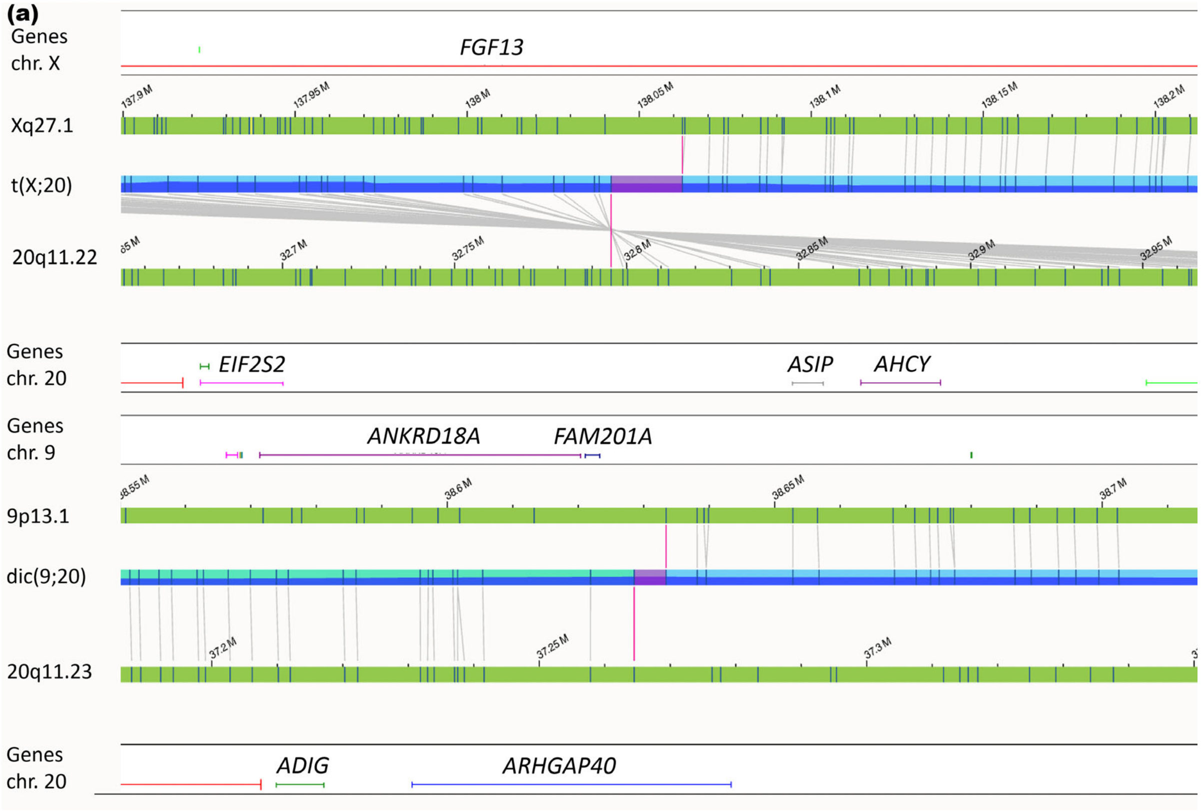

Figure S1. Translocations in patient 4 by means of OGM. The upper green bars indicate the reference maps. The maps of the patient are displayed in blue/turquoise; the breakpoints are marked with red lines. Genes located within the shown regions are given above or below. **(a)** Translocation t(X;20)(q27.1;q11.22), half of the patient map fits to chromosome chr Xq27.1, the other half fits to chr 20q11.22. The breakpoints occur in *FGF13* on chr X and in an intergenic region on chr 20. **(b)** Translocation dic(9;20)(p13.1;q11.23) half of the patient map fits to chromosome chr 9p13.1, the other half fits to chr 20q11.23. The breakpoints occur in an intergenic region on chr 9 and in *ARHGAP40* on chr 20.

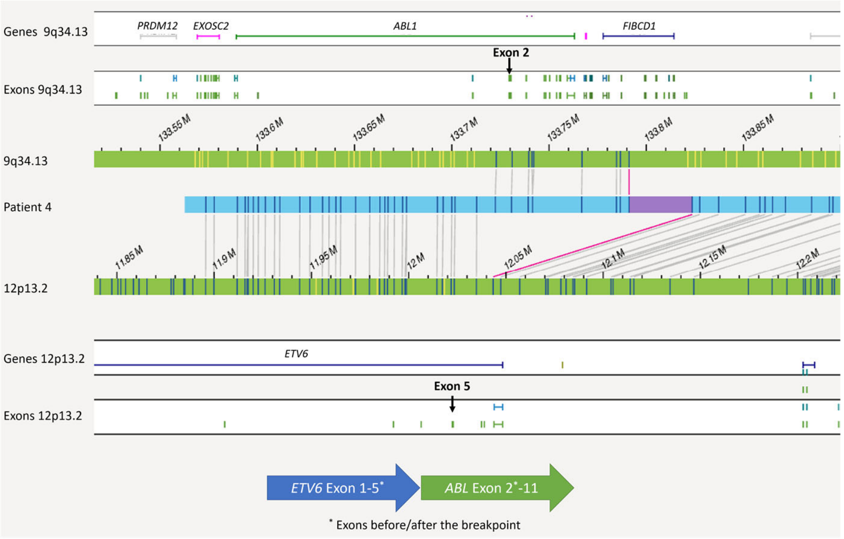

Figure S2. Insertion (12;9) in patient 4 by means of OGM. The green bars indicate the reference maps. The map of the patient is displayed in blue. Genes and Exons located within the shown regions are given above or below. Genomic material from chromosome 9 (133722840_133791173) containing exon 2-11 of the *ABL* gene is inserted in chromosome 12 between position 12034841 and 12043458. The insertion is leading to a fusion of *ETV6* and *ABL1* schematically shown

below. Due to the label distribution, it is also possible that the fusion gene contains exon 6 and/or exon 7 of *ETV6*. The *de novo* assembly algorithm detected the insertion, however, the RVP was used to identify the inserted material.

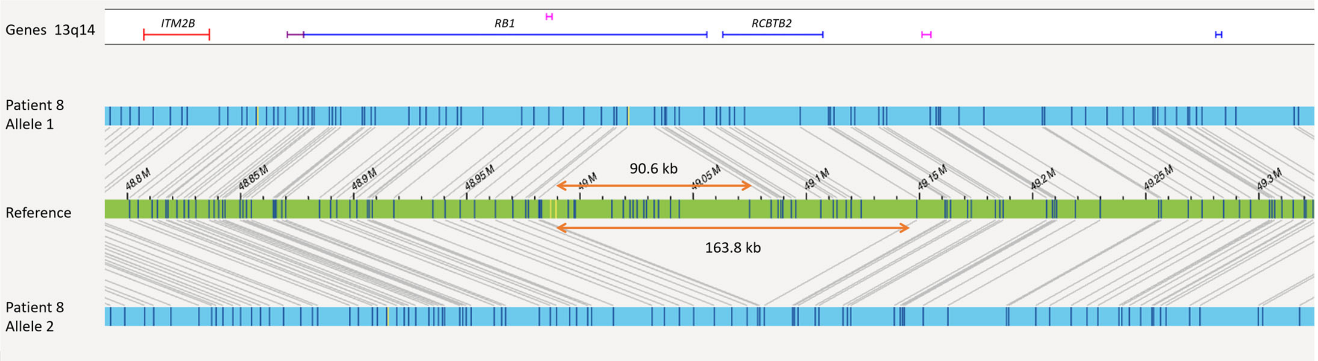

Figure S3. Bi-allelic deletion in *RB1* (chr 13q14) in patient 8 by means of OGM. The green bar indicates the reference map. The maps of the patient are displayed in blue. Genes located within the shown regions are given above or below. The overlapping deletions targeting *RB1* are 90.6 kb and 163.8 kb large and marked with a red arrow.

Table S1. Quality report of de novo assemblies.

Sample	1	2	3	4	5	6	7	8	9	10	11	12	Total
Sample storage	Banked	Banked	Banked	Banked	Banked	Fresh	Fresh	Fresh	Fresh	Fresh	Fresh	Fresh	-
Cell count [$\times 10^6$]	72	20	27	25	24	8.7	81	45	87	82	6.9	61	≥6.9 (45±30)
Blasts [%]	92.5	91.0	88.0	94.0	83.0	77.7	96.1	50.3	91.0	75.8	83.5	79.8	≥50.3 (83.6±12.4)
Total number of molecules 150 kb [$\times 10^6$]	1.42	1.52	1.52	1.48	1.93	1.94	1.91	1.55	1.60	1.35	2.05	1.95	≥1.35 (1.68±0.25)
Total length [Gb]	430	426	470	473	486	446	438	400	458	331	454	455	≥372 (439±41)
Average length [kb]	302	280	310	319	252	230	229	258	287	244	221	233	≥221 (264±34)
Molecule N50 [kb]	322	294	331	343	253	224	226	258	295	244	216	232	>216 (270±45)
Average label density [per 100 kb]	15.6	15.7	15.5	15.6	15.5	15.6	15.3	15.6	15.2	15.7	15.7	15.8	15.2-15.8 (15.6±0.2)
Coverage of the reference [×hg19]	139	138	152	153	157	144	141	120	148	107	147	147	≥107 (141±14)
Total number of molecules aligned [$\times 10^6$]	1.29	1.40	1.41	1.38	1.77	1.75	1.71	1.28	1.49	1.23	1.78	1.77	≥1.23 (1.52±0.22)
Fraction of molecules aligned	0.91	0.92	0.93	0.93	0.92	0.91	0.90	0.87	0.93	0.91	0.87	0.91	≥0.87 (0.91±0.02)
Effective coverage of the reference [×hg19]	114	115	125	126	129	109	104	92	115	84	106	116	≥84 (111±13)
Average confidence	40.9	39.5	44.0	46.2	34.6	33.1	33.0	33.4	41.7	36.6	31.2	33.9	≥31.2 (37.3±4.9)
Diploid genome map count	421	436	435	414	402	552	508	423	429	529	504	488	≥402 (462±51)
Diploid genome map length [Gb]	5.84	5.81	5.88	5.81	5.81	5.84	5.81	5.81	5.78	5.85	5.79	5.83	≥5.78 (5.82±0.03)
Diploid genome map N50 [Mb]	70.2	69.8	59.7	79.9	59.8	58.2	59.6	69.6	58.1	58.5	58.5	58.5	≥58.1 (63.4±7.2)
Haploid genome map count	263	286	275	265	250	367	336	272	274	338	337	318	≥250 (298±38)
Haploid genome map length [Gb]	2.98	3.01	2.98	3.01	3.09	3.12	3.07	3.02	2.99	3.10	3.12	2.98	≥2.98 (3.04±0.06)
Haploid genome map N50 [Mb]	66.5	62.9	59.7	73.5	58.4	58.4	58.5	59.6	57.3	55.8	56.8	58.5	≥55.8 (60.5±5.0)

Total reference length [Gb]	3.10	3.10	3.10	3.10	3.10	3.10	3.10	3.10	3.10	3.10	3.10	3.10	3.10 (3.10±0.00)
Total number of genome maps aligned (fraction)	362 (0.86)	362 (0.83)	355 (0.82)	336 (0.81)	331 (0.82)	455 (0.82)	403 (0.79)	334 (0.79)	358 (0.83)	424 (0.80)	399 (0.79)	386 (0.79)	≥331 (≥0.79) (375±39 ((0.81±0.02))
Total unique aligned length [Gb]	2.89	2.86	2.86	2.84	2.84	2.84	2.86	2.84	2.83	2.86	2.84	2.86	≥2.83 (2.85±0.02)
Total unique aligned length [per reference length]	0.92	0.92	0.92	0.92	0.92	0.92	0.92	0.92	0.92	0.92	0.92	0.92	≥0.92 (0.92±0.00)
Total number of molecules aligned [$\times 10^6$]	1.31	1.42	1.43	1.41	1.80	1.77	1.73	1.30	1.51	1.25	1.80	1.80	≥1.25 (1.54±0.22)
Fraction of molecules aligned	0.920	0.936	0.943	0.950	0.932	0.914	0.909	0.888	0.943	0.920	0.975	0.923	≥0.888 (0.929±0.022)
Effective coverage of assembly [x]	74.1	76.6	82.0	83.3	88.7	73.7	71.5	61.8	77.1	55.6	74.0	79.5	≥55.6 (74.8±9.0)
Average confidence	54.1	52.5	58.2	60.0	47.2	45.4	45.4	45.0	55.6	48.4	43.2	46.2	≥43.2 (50.1±5.7)
Deletions	1377	1312	1341	1336	1311	1343	1372	1339	1341	1357	1338	1269	1336±29
Insertions	2862	2843	2828	2896	2787	2819	2784	2831	2784	2778	2748	2785	2812±42
Duplications	40	47	44	43	29	41	34	36	39	38	29	54	40±7
Inversion breakpoints	90	90	81	94	93	94	77	96	87	74	70	72	85±10
Interchr. translocation breakpoints	2	1	2	2	0	27	29	0	3	3	0	5	6±10
Intrachr. translocation breakpoints	0	1	0	0	0	44	23	0	1	0	3	2	6±14
